# 慢病毒载体介导*IL-24*基因转导人骨髓间充质干细胞的实验研究

**DOI:** 10.3779/j.issn.1009-3419.2013.01.02

**Published:** 2013-01-20

**Authors:** 秋干 祁, 清华 周, 印 李, 伟强 李, 霏 陈, 建军 秦

**Affiliations:** 1 300052 天津，天津市肺癌转移与肿瘤微环境重点实验室，天津市肺癌研究所，天津医科大学总医院 Tianjin Key Laboratory of Lung Cancer Metastasis and Tumor Microenvironment, Tianjin Lung Cancer Institute, Tianjin Medical University General Hospital, Tianjin 300052, China; 2 450005 郑州，郑州大学附属肿瘤医院胸外科 Department of Thoracic Surgery, Tumor Hospital of He'nan, Zhengzhou 450005, China; 3 510080 广州，中山大学干细胞与组织工程中心 Center for Stem Cell Biology and Tissue Engineering, Sun Yat-sen University, Guangzhou 510080, China

**Keywords:** 人骨髓间充质干细胞, IL-24, EGFP, 基因转导, 慢病毒载体, hBMSCs, IL-24, EGFP, Gene transduction, Lentiviral vector

## Abstract

**背景与目的:**

以人骨髓间充质干细胞（human bone marrow mesenchymal stem cells, hBMSCs）作为抑癌基因*IL-24*的细胞载体的研究目前未见报道。应用Gateway法构建共表达增强型绿色荧光蛋白（enhanced green fluorescent protein, *EGFP*）基因和*IL-24*基因的慢病毒载体，探讨其对hBMSCs的转导情况，为今后肿瘤的基因治疗奠定基础。

**方法:**

应用DNA重组技术构建含有*IL-24*和*EGFP*基因的慢病毒表达载体，并与慢病毒包装系统（ViraPower^TM^ Lentiviral Packaging Mix）共转染293FT细胞，收集上清，纯化浓缩，测定重组病毒的滴度。取重组慢病毒感染hBMSCs，通过嘌呤霉素筛选并纯化hBMSCs，应用实时荧光定量PCR（quantitative PCR, qPCR）及ELISA法分别检测hBMSCs中IL-24 mRNA及IL-24蛋白水平的表达情况。

**结果:**

成功构建了共表达*IL-24*和*EGFP*基因的重组慢病毒载体，经包装、纯化及浓缩，病毒滴度为7.25×10^7^ PFU/mL。重组慢病毒转导hBMSCs后，通过筛选获得纯化，转导效率可达到100%。qPCR检测示：转导组IL-24 mRNA表达明显高于未转导组（*P* < 0.05）; ELISA法检测显示转导组hBMSCs上清液IL-24蛋白表达40 μg/L，未转导组为阴性。

**结论:**

构建的携带*IL-24*基因的重组慢病毒载体可有效转导hBMSCs，表达IL-24蛋白。

## Abstract (en)

**Background and objective:**

Up to know, no any study on using human bone marrow mesenchymal stem cells (hBMSCs) as cells carrier of tumor suppressor gene (*IL-24*) was reported. The aim of this study is to study the efficiency of transduction of hBMSCs by constructing the lentiviral vector in co-expressing enhanced green fluorescent protein (*EGFP*) gene and human *IL-24* gene, and to lay a foundation for gene therapy of tumor in the future.

**Methods:**

The lentivector which contain IL-24 and EGFP constructed by recombinant DNA technology were co-transfected to 293FT cells with ViraPower^TM^ Lentiviral Packaging Mix. The recombinant lentivirus infected with hBMSCs were selected and purified by puromycin. Expression of IL-24 mRNA and IL-24 protein levels were detected by real-time quantitative PCR (qPCR) and ELISA.

**Results:**

The recombinant lentiviral vector of co-expressing *IL-24* gene and *EGFP* gene were successfully constructed by multisite Gateway technology, virus can be packaged, purified and concentrated successfully, and the virus titer was 7.25×10^7^ PFU/mL. The efficiency of recombinant lentivirus to transduce hBMSCs can reach 100% after selection. The result of qPCR showed that the level of IL-24 mRNA expression in transduced group was significantly higher than that in non-transduced group (*P* < 0.05); ELISA detection confirmed that IL-24 protein expression of transduced group was positive in supernatant and the concentration of IL-24 protein is 40 μg/L, while the non-transduced group was negative.

**Conclusion:**

Lentiviral vector carrying recombinant *IL-24* gene can effectively transduce hBMSCs and express IL-24 protein.

肿瘤的复发与转移是肿瘤治疗失败的主要原因，能准确发现肿瘤的微卫星灶，并将治疗药物靶向作用于肿瘤部位，是治疗肿瘤直接而有效的途径。近来研究^[1]^发现干细胞具有向肿瘤定向迁移的特性，利用干细胞为载体携带治疗基因或药物靶向治疗肿瘤可能是肿瘤治疗的新突破。人骨髓来源的间充质细胞（human bone marrow mesenchymal stem cells, hBMSCs）具有在不同的诱导条件下向内胚层、中胚层和外胚层的所有类型细胞分化的潜能^[2]^，而且取材方便、体外分离与增殖技术成熟等特点，在干细胞移植和基因治疗中颇受青睐^[3, 4]^。

白细胞介素-24（interleukin-24, IL-24）又称黑色素瘤分化相关基因-7（melanoma differentiation associated gene 7, MDA-7）是又一个既能抑制肿瘤细胞生长和血管形成并诱导凋亡，同时又能刺激免疫细胞表达细胞因子的新型抑癌基因^[5, 6]^。选择性清除肿瘤细胞，而不损伤正常细胞，是最广泛的“靶向”概念。肿瘤基因治疗成功的关键在于有效基因载体的选择，可以携带治疗因子释放到相应的靶器官而发挥生物学效应。

实验基于以上研究背景，通过多位点Gateway技术^[7]^构建了携带*IL-24*基因的慢病毒表达载体pLVpuro/EF1-IL-24-IRES-EGFP，将其包装成慢病毒，转导入hBMSCs，使用嘌呤霉素进行筛选纯化，以获得过表达外源基因*IL-24*及报告基因*EGFP*的绿色荧光细胞，利用*EGFP*基因实现目的基因在体内外表达的示踪定位，为下一步开展基因治疗相关研究奠定基础。

## 1 材料与方法

### 1.1 材料

带启动子的入门克隆pUp-EF1α、带报告基因的入门克隆pTail-IRES-EGFP、目的载体pDEST-puromycin、入门载体pDONR^TM^221、ViraPower^TM^ Lentiviral Packaging Mix、BP Clonase^TM^Ⅱ和LR Clonase^TM^Ⅱ、Lipofectamine^TM^2000、OneShot^®^ Stbl3 Chemically Competent E. coli、239FT细胞株、CD分子单抗（CD29-APC、CD90-PE、CD34-FITC、CD45- FITC）均购自美国Invitrogen公司，hBMSCs由中山大学干细胞中心提供，CDS克隆（含*IL-24*基因）由中山大学干细胞中心项鹏教授馈赠，重组人IL-24 ELISA试剂盒购自美国RD公司，胎牛血清及L-DMEM均购自Gibco公司。

### 1.2 实验方法

#### 1.2.1 入门克隆pDown-IL-24的构建和鉴定

引物设计：根据Genebank中*IL-24*基因（NM_006850）的cDNA序列，设计IL-24引物序列为：Forward: 5’-GGGG ACAAGTTTGTACAAAAAAGCAGGCTCCATCGATGGATGAATTTTCAACAGAGGCTG-3’; Reverse: 5’-GGGGACCACTTTGTACAAGAAAGCTGGGTACGGGATCCTCAGAGCTTGT AGA ATTTC-3’。PCR扩增：在上述引物的作用下，以IL-24的cDNA为模板进行扩增，电泳检测PCR产物，切胶回收纯化目的条带。BP重组反应：用侧翼含attB位点的PCR产物与入门载体pDONR^TM^221进行重组反应。取2 μL反应产物转化50 μL Stbl3感受态细胞。细胞涂布于含有50 mg/L卡那霉素的LB平板，37 ℃过夜培养。鉴定：从平板挑单克隆接种于LB液体培养液中，37 ℃、220 r/min振摇过夜培养，菌液行PCR扩增验证，挑取构建正确的菌落培养过夜，提取质粒DNA，测序验证。

#### 1.2.2 表达克隆pLVpuro/EF1α-IL-24-IRES-EGFP的构建和鉴定

将入门克隆pUp-EF1α、pDown-IL-24和pTail-IRES-EGFP与目的载体pDEST-puromycin在LR重组反应酶作用下重组，取2 μL重组反应液转化50 μL Stbl3感受态细胞，氨卞西林LB培养平板筛选阳性重组克隆并扩增，菌液行PCR扩增验证，挑取构建正确的菌落培养过夜，提取质粒DNA，测序验证。

#### 1.2.3 慢病毒的包装和浓缩

将慢病毒包装质粒ViraPower^TM^ Lentiviral Packaging Mix和载体质粒pLVpuro/EF1α-IL-24-IRES-EGFP以1:1的摩尔比例混合, 在Lipofectamine^TM^ 2000的作用下共转染293FT细胞，置于37 ℃、5%CO_2_的培养箱中培养。12h后换液，加入新鲜293FT细胞培养液。72h后观察有强绿色荧光及细胞融合现象时收集培养上清，4 ℃、4, 500 r/min离心15 min，将病毒上清液用0.45 μm滤膜过滤以去除细胞碎片，4 ℃、50, 000g高速离心90 min。用RNase-free L-DMEM悬浮病毒颗粒沉淀，标记LV-IL-24，-80 ℃冻存备用。同法构建只含EGFP的空病毒，标记LV-EGFP。

#### 1.2.4 慢病毒的滴度测定

接种293FT细胞于6孔板，每孔接种2×10^5^个细胞，37 ℃培养过夜; 第2天，将慢病毒溶液用无血清DMEM（不含抗生素）按1×10^-2^、1×10^-3^、1×10^-4^、1×10^-5^、1×10^-6^、1×10^-7^进行系列稀释，将其分别加入到相应的孔中，加入聚酰胺（Polybrene）终浓度至6 mg/L，置37 ℃、5%CO_2_培养箱培养。12 h后全量换液，加入新鲜的293FT培养液。第4天观察荧光表达情况，荧光细胞数随稀释倍数增加而减少，计数出表达荧光的细胞个数，将得到的数值乘以相应的稀释倍数就得到病毒原液的滴度数。

#### 1.2.5 hBMSCs培养及鉴定

常规复苏冻存的hBMSCs，用含10%胎牛血清的L-DMEM培养液，置于置37 ℃、5%CO_2_的培养箱培养，每2-3天常规换液。待细胞长至80%-90%融合时，用0.25%胰蛋白酶消化传代，传代后约1周左右达90%融合。免疫组化及流式细胞术鉴定细胞表面标志物CD29、CD90、CD34和CD45。

#### 1.2.6 慢病毒转导hBMSCs及纯化

用慢病毒LV-IL-24及LV-EGFP进行转导传代后状态较好的hBMSCs，设单纯hBMSCs为对照。分别加入病毒悬液20 μL和Polybrene（终浓度6 mg/L），置37 ℃、5%CO_2_的培养箱中培养。12 h后去除含病毒的培养液，换为L-DMEM培养液。连续感染3次后用L-DMEM培养液继续培养。转导后为了得到纯化的感染细胞群，在转导后第7天开始使用1 mg/L-5 mg/L嘌呤霉素进行筛选，筛选时间为7 d-10 d。纯化后转入*IL-24*基因的hBMSCs标记为hBMSCs-IL-24;转入*EGFP*基因的hBMSCs标记为hBMSCs-EGFP。

#### 1.2.7 转导纯化后的hBMSCs的鉴定分析

实时荧光定量PCR（quantitative PCR, qPCR）检测：收集hBMSCs-IL-24组、hBMSCs-EGFP组及对照组hBMSCs细胞，提取各组RNA，反转录cDNA，然后以cDNA为模板，行qPCR检测各组中IL-24 mRNA的表达水平。ELISA检测：收集hBMSCs-IL-24组、hBMSCs-EGFP组及对照组hBMSCs细胞培养液上清，根据重组人IL-24 ELISA试剂盒操作说明检测各组细胞的IL-24蛋白浓度水平。

### 1.3 统计学分析

采用SPSS 13.0统计学软件对IL-24评分结果进行单因素方差分析（*one-way* *ANOVA*），*P* < 0.05为差异有统计学意义。

## 2 结果

### 2.1 入门克隆pDown-IL-24的鉴定

经BP重组反应，挑选两个入门克隆阳性菌落，质粒PCR扩增片段大小在600 bp-700 bp之间，与*IL-24*基因理论值一致（621 bp），见图 1。挑取构建正确的菌落培养过夜后提取质粒DNA，经测序鉴定目的基因序列与数据库的*IL-24*基因序列吻合，表明成功构建入门克隆（pDown-IL-24）。

**1:**
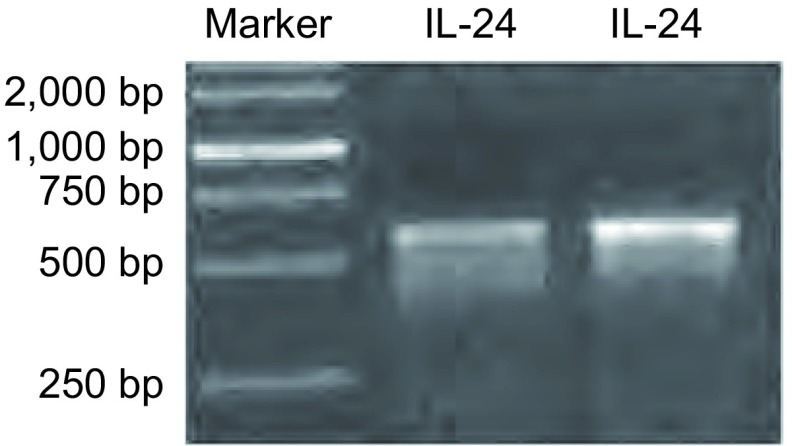
入门克隆PCR鉴定结果 PCR identification results of entry clone

### 2.2 表达克隆pLVpuro/EF1α-IL-24-IRES-EGFP的构建和鉴定

经LR重组得到了表达克隆阳性菌落，提取克隆提取质粒行PCR扩增出目的片段包括表达基因*IL-24*（621 bp）、启动子EF1α（1, 264 bp）和报告基因*EGFP*（750 bp），结果与基因片段大小相符（图 2）。

**2:**
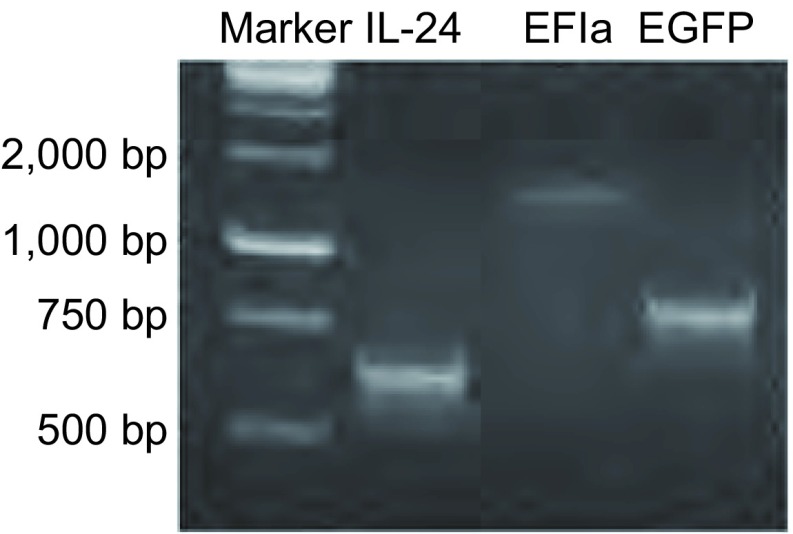
表达克隆PCR鉴定结果 PCR identification results of expression cloning

### 2.3 慢病毒的包装与浓缩

慢病毒载体共同转染293FT细胞72 h后，在荧光显微镜下观察，转*IL-24*基因组及空病毒载体组均可见大量强绿色荧光，证实质粒转染入293FT细胞，细胞部分融合，可见多核复合体出现（图 3）。收集病毒上清液高速离心后，用100 μL RNase-free L-DMEM分别溶解病毒颗粒。

**3:**
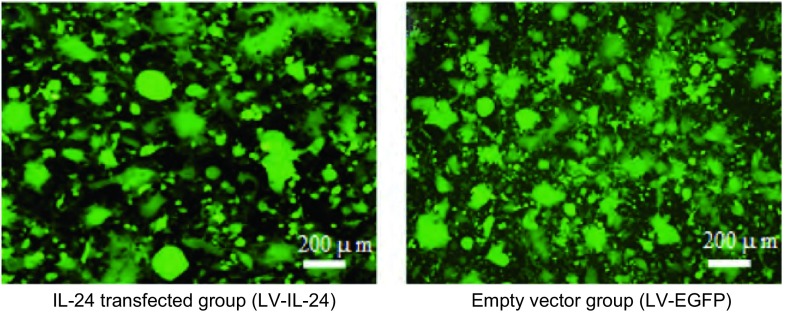
转染72 h的293FT细胞绿色荧光 The green fluorescent of 293 FT cells after transfection for 72 h

### 2.4 慢病毒的滴度测定

将病毒原液（LV-IL-24）从1×10^-2^到1×10^-7^梯度稀释后，感染293FT细胞2 d后，可见1×10^-2^到1×10^-5^孔均出现大量阳性细胞，而1×10^-6^到1×10^-7^孔阳性细胞数分别为45个和10个，根据公式病毒滴度=阳性细胞数×病毒上清稀释倍数，取1×10^-6^到1×10^-7^孔两孔平均值得出LV-IL-24病毒滴度为：7.25×10^7^ PFU/mL; 同法测空病毒（LV-EGFP）滴度为8.12×10^7^ PFU/mL。

### 2.5 hBMSCs培养及鉴定

常规复苏hBMSCs后约7 d到达90%融合，细胞的排列呈典型的漩涡样，单个细胞呈典型纺锤型。经1:3比例传代之后，细胞经3 d-4 d培养可以达到融合90%以上并适于再次传代。流式细胞术分析结果显示：hBMSCs均一性可达95%以上，hBMSCs高表达CD29、CD90，不表达DCD34、CD4（图 4）。

**4:**
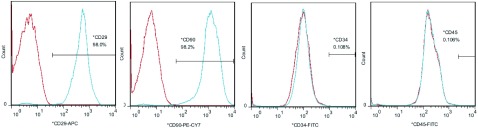
人骨髓间充质干细胞（human bone marrow mesenchymal stem cells, hBMSCs）表面抗原标志 Surface antigen mark of hBMSCs

### 2.6 慢病毒载体转导纯化后hBMSCs绿色荧光观察

慢病毒（LV-IL-24, LV-EGFP）转染hBMSCs 72 h后，荧光显微镜下观察到部分细胞表达绿色荧光。7 d后，镜下可见绿色荧光细胞明显增多，此时开始加入嘌呤霉素进行筛选，维持时间为7 d-10 d，期间细胞常规传代，筛选结束后，即可得到携带慢病毒载体的绿色荧光细胞群（图 5）。

**5:**
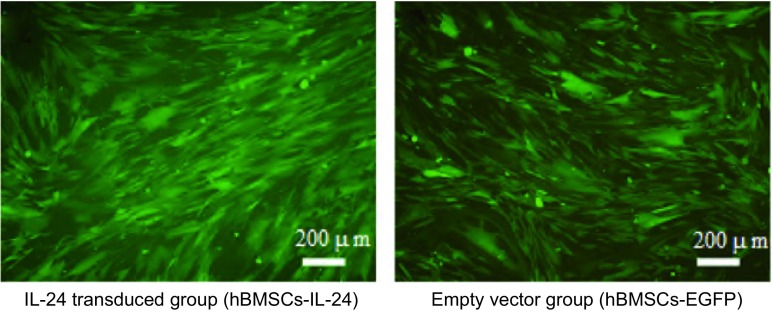
慢病毒转导纯化后hBMSCs的绿色荧光表达情况 The green fluorescent after lentivirus transducs hBMSCs

### 2.7 转导纯化后的hBMSCs的鉴定分析

qPCR检测结果：hBMSCs组与hBMSCs-EGFP组可检测到IL-24 mRNA少量表达，而hBMSCs-IL-24组可检测到IL-24 mRNA明显表达（*P* < 0.05）（图 6）。ELISA检测结果：hBMSCs组与hBMSCs-EGFP组中几乎检测不到IL-24的浓度水平，hBMSCs-IL-24组中IL-24的浓度可到达40 μg/L，表明*IL-24*基因成功转入到hBMSCs中。

**6:**
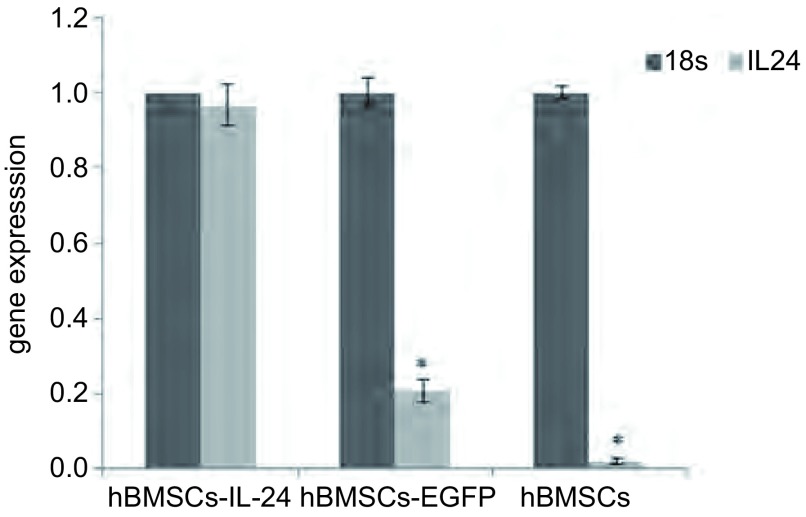
qPCR检测IL-24 mRNA表达。^*^: *P* < 0.05 Expression of IL-24 mRNA detected by qPCR. ^*^: *P* < 0.05

## 3 讨论

hBMSCs是具有多向分化潜能的干细胞，在体外能贴壁培养和大量扩增，hBMSCs属成体干细胞，不仅可以向中胚层分化，在特定条件下，还能实现跨系统分化^[8, 9]^，其植入体内无免疫排斥反应，并且能广泛迁移，易于组织整合而发挥功能，都提示其在基因治疗中可能成为非常有价值的运载细胞^[10-12]^。而且多项研究^[1, 13]^表明，hBMSCs在体内有向肿瘤微环境趋向转移的特性。将hBMSCs作为细胞载体开展肿瘤的基因治疗是目前研究的热点之一。

慢病毒属于逆转录病毒的一种^[14]^，可以高效感染处于分裂期和非分裂期的细胞; 借助慢病毒可将其携带的外源基因高效整合进宿主细胞基因组中，外源基因可在细胞内长期稳定表达。应用Gateway技术构建慢病毒载体已得到广泛应用^[15]^，明显简化了基因克隆和亚克隆的步骤，避免了假阳性的出现，使基因克隆更加方便易行。

hBMSCs具有取材方便、体外易培养、强大的迁移能力及高效安全的外源基因表达能力等优点，故被认为是组织工程和基因工程理想的靶细胞^[16, 17]^。选择构建以慢病毒为载体的表达系统，对于较难转染的干细胞，能明显提高目的基因转导效率，该载体可以将外源基因有效地整合到宿主染色体上，从而达到持久性表达^[18]^。本实验利用Gateway技术成功构建了慢病毒表达载体（pLVpuro/EF1α-IL-24-IRES-EGFP），将其包装成慢病毒，同时将携带有目的基因的慢病毒成功转导hBMSCs，编码出来的绿色荧光蛋白和目的基因蛋白不是融合蛋白，而是各具独立空间结构和生理活性，有利于目的基因蛋白的表达鉴定及定位追踪^[19]^，同时为利用干细胞作为细胞载体携带治疗基因或药物靶向治疗肿瘤提供新的理论依据。

## References

[1] Kidd S, Spaeth E, Dembinski JL (2009). Direct evidence of mesenchymal stem cell tropism for tumor and wounding microenvironments using *in vivo* bioluminescent imaging. J Stem Cells.

[2] Da Silva ML, Chagastelles PC, Nardi NB (2006). Mesenchymal stem cells reside in virtually all post-natal organs and tissues. Cell Sci.

[3] Bexell D, Scheding S, Bengzon J (2010). Toward brain tumor gene therapy using multipotent mesenchymal stromal cell vectors. J Mol Ther.

[4] Sun XY, Nong J, Qin K (2011). Mesenchymal stem cell-mediated cancer therapy: A dual-targeted strategy of personalized medicine. World J Stem Cells.

[5] Lebedeva IV, Emdad L, Su ZZ (2007). mda-7/IL-24, novel anticancer cytokine: focus on bystander antitumor, radiosensitization and antiangiogenic properties and overview of the phase Ⅰ clinical experience. Int J Oncol.

[6] Emdad L, Lebedeva IV, Su ZZ (2009). Historical perspective and recent insights into our understanding of the molecular and biochemical basis of the antitumor properties of mda-7/IL-24. J Cancer Biol Ther.

[7] Petersen LK, Stowers RS (2011). A Gateway MultiSite recombination cloning tool kit. PLoS One.

[8] Da Silva ML, Chagastelles PC, Nardi NB (2006). Mesenchymal stem cells reside in virtually all post-natal organs and tissues. Cell Sci.

[9] Dominici M, Le Blanc K, Mueller I (2006). Minimal criteria for defining multipotent mesenchymal stromal cells. The International Society for Cellular Therapy position statement. Cytotherapy.

[10] Okada T, Ozawa K (2008). Vector-producing tumor-tracking multipotent mesenchymal stromal cells for suicide cancer gene therapy. J Front Biosci.

[11] Okada T (2010). Gene therapy with vector-producing multipotent mesenchymal stromal cells. J Yakugaku Zasshi.

[12] Song F, Xing Q, Song KD (2012). The antitumor effect of mesenchymal stem cells transduced with a lentiviral vector expressing cytosine deaminasein a rat glioma model. J Cancer Res Clin Oncol.

[13] Beckermann BM, Kallifatidis G, Groth A (2008). VEGF expression by mesenchymal stem cells contributes to angiogenesis in pancreatic carcinoma. Br J Cancer.

[14] Pacchia AL, Adelson ME, Kaul M (2001). An inducible packaging cell system for safe, efficient lentiviral vector production in the absence of HIV-1 accessory proteins. J Virol.

[15] Li W, Liu C, Qin J (2010). Efficient genetic modification of cynomolgus monkey embryonic stem cells with lentiviral vectors. J Cell Transplant.

[16] Gao P, Ding Q, Wu Z (2010). Therapeutic potential of human mesenchymal stem cells producing IL-12 in a mouse xenograft model of renal cell carcinoma. Cancer Lett.

[17] Wei HJ, Wu AT, Hsu CH (2011). The development of a novel cancer immunotherapeutic platform using tumor-targeting mesenchymal stem cells and a protein vaccine. J Mol Ther.

[18] Lu XF, Du JC, Li L (2011). Construction of modular lentivector and its application in transduction of human mesenchymal stem cells. Zhongguo Zu Zhi Gong Cheng Yan Jiu Yu Lin Chuang Kang Fu.

[19] Lavender JS, Kinzelman JL (2009). A cross comparison of QPCR to agar-based or defined substrate test methods for the determination of Escherichia coli and enterococci in municipal water quality monitoring programs. J Water Res.

